# Optimized Schwarz waveform relaxation methods for wave-heat coupling in one dimensional bounded domains

**DOI:** 10.1007/s11075-025-02100-1

**Published:** 2025-05-27

**Authors:** Franz Chouly, Martin J. Gander, Véronique Martin

**Affiliations:** 1 Center of Mathematics, University of the Republic, Montevideo, Uruguay; 2 Section de Mathématiques, Université de Genève, Genève, Suisse; 3 Laboratoire Amiénois de Mathématique Fondamentale et Appliquée UMR CNRS 7352, Amiens, France

**Keywords:** Heterogeneous domain decomposition methods, Optimized Schwarz waveform relaxation

## Abstract

We are interested in heterogeneous domain decomposition methods to couple partial differential equations in space-time. The coupling can be used to describe the exchange of heat or forces or both, and has important applications like fluid-structure or ocean-atmosphere coupling. Heterogeneous domain decomposition methods permit furthermore the reuse of existing codes which represent long term investments, a further great advantage in applications. We require that our method can use different and adaptive time steps for the different models, can be executed in parallel, is robust, and can use independent fast inner solvers. An ideal candidate is Optimized Schwarz Waveform Relaxation (OSWR) that can be used without overlap, which is important for the different physical models. We focus here on the model problem of coupling a heat and a wave equation in one spatial dimension, which we consider to be a minimal example of relevance, and our goal is to design and analyze transmission conditions such that OSWR converges as fast as possible. We propose two strategies, a first one where we optimize the transmission using one common parameter, and a second one where we use the wave characteristics of one subdomain to choose one parameter, and then optimize the other. We illustrate our results with numerical experiments.

## Introduction

Heterogeneous domain decomposition methods are a very active field of research, for a brief introduction, see [1]. Since Optimized Schwarz Methods (OSMs) can be used with non-overlapping subdomains, see  [2], they are ideal candidates for heterogeneous domain decomposition where the physics in different domains is different and requires different numerical treatment with possibly different codes. In addition, in OSMs one optimizes transmission conditions between subdomains for fast convergence, and OSMs can even take advantage of the different physics to converge faster than if the physical properties were the same, see [3] for a typical example of diffusion with jumping coefficients.

There has been substantial progress in heterogeneous domain decomposition for steady problems, see for example [4] for Helmholtz-Laplace coupling, [5] for the coupling of different elliptic partial differential equations, [6–9] for Stokes-Darcy coupling, [10] for time discretized fluid-structure interaction in cylindrical geometry, [11] for a corresponding stationary case in spherical geometry, and [12] for stationary porous medium equations coupled with Navier-Stokes. As alternative, one can also use more monolithic approaches for fluid-structure interaction, see e.g. [13–17] and references therein, but the reuse of existing codes is more difficult then.

More recently, also time dependent heterogeneous domain decomposition methods were proposed and analyzed, see [18, 19] where the heterogeneity in the models comes from the need of computational savings, [20, 21] where Dirichlet-Neumann Waveform Relaxation methods were studied, and [22, 23] with continuous and discrete analyses of SWR for a reaction diffusion problem with jumping coefficients. In [24], an Optimized Schwarz Waveform Relaxation algorithm (OSWR) was studied for a heat-wave coupling in 1D on unbounded domains, which is a minimal example of relevance for fluid-structure interaction [25]. This type of parabolic-hyperbolic coupling appears in many fluid-structure interaction phenomena and lies at the heart of many applications, where the viscous fluid acts as a parabolic operator, while the elastic structure acts as a second order hyperbolic operator. Coupling conditions ensure the continuity of velocity and stresses at the interface between the fluid and the solid. Fluid-structure interaction has its origins in aerospace/aeronautics [26, 27] and nuclear energy production [28–30], but nowadays it covers many more applications, for instance in bio-mechanics for blood flows [31–34] and respiratory flows [16, 35]. It is also involved in industrial applications such as wind energy production [36] or parachute simulation [37]. General monographs on fluid-structure interaction are for instance [38, 39]. On unbounded domains in 1D, optimal transmission conditions for OSWR turn out to be particularly simple for the wave equation domain [24], since the best transmission condition choice is still local, see [40] for OSWR for wave equations, and [41] for a general discussion. The best transmission condition for the heat domain still involves a non-local operator [42], see also [43] for the specific case of the 1D heat equation.

Changing to bounded domains has however a fundamental influence on the performance of such algorithms for the wave equation domain and hyperbolic problems in general, which was only recently discovered for the time harmonic case, see [44]. We therefore study here for the first time the relevant heat-wave coupling problem and associated OSWR algorithms for the case of bounded domains with Robin, Dirichlet or Neumann boundary conditions on the external boundaries. This is closer to practical applications in which fluid and solid domains are bounded and displacements, velocities and tractions are imposed on the external boundaries, see, *e.g.*, [45]. We derive optimized conditions that take into account the size of each subdomain and the external boundary conditions. The optimal transmission conditions in this situation are more complicated than those of [24], and require approximations for practical use, leading to an optimization process for best performance. We propose here two new such approximations, a first one where both the heat and the wave domain use the same optimized parameter, and a second one where we use for the wave domain a local optimal parameter, and then optimize the heat parameter for this setting.

## Heat-Wave coupled model problem

**Fig. 1:**
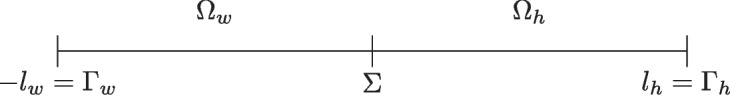
Wave domain $\Omega _w$, heat domain $\Omega _h$, and interface $\Sigma$

Let $l_w>0$ and $l_h>0$ be the domain length of the wave and heat domains, $\Omega _w:= (-l_w,0)$ and $\Omega _h:= (0,l_h)$, and let $\Sigma := \overline{\Omega _w} \cap \overline{\Omega _h} = \{0\}$ be the interface, see Fig. 1.

We denote the outer physical boundaries by $\Gamma _h= \{l_h\}$ and $\Gamma _w = \{-l_w\}$. We are interested in designing and studying a heterogeneous OSWR algorithm for the heat and wave coupled problem: Find $v : {[0,T]} \times \Omega _w\rightarrow \mathbb {R}$ and $u : {[0,T]} \times \Omega _h\rightarrow \mathbb {R}$ such that

1 $$\begin{aligned} \begin{aligned}&\left\{ \begin{aligned}&\partial ^2_{t}{v} - c^2 \, \partial ^2_{x}{v} = f  &   \text {in}\ {[0,T]}\times \Omega _w, \\&-{{\partial _{x}}} v + \alpha _w \, v = 0  &   \text {on} \ {[0,T]}\times \Gamma _w, \\&v (0,\cdot ) = v_{0}  &   \text {in}\ \Omega _w, \\&\partial _tv (0,\cdot ) = \dot{v}_{0}  &   \text {in}\ \Omega _w, \\ \end{aligned} \right. \\&\left\{ \begin{aligned}&\partial _t{u} - \kappa \, \partial ^2_{x}{u} = g  &   \text {in}\ {[0,T]}\times \Omega _h, \\&{{\partial _{x}}u} + \alpha _h \, u = 0  &   \text {on}\ {[0,T]}\times \Gamma _h,\\&u(0,\cdot ) = u_0  &   \text {in}\ \Omega _h, \\ \end{aligned} \right. \end{aligned} \end{aligned}$$

together with the coupling conditions at the interface $\Sigma$

2 $$\begin{aligned} \begin{aligned}&\left\{ \begin{aligned}&\partial _tv = u  &   \text {on}\ {[0,T]}\times \Sigma , \\&c^2 {\partial _{x}}{v} {- \kappa \, {\partial _{x}}{u}} = 0  &   \text {on}\ {[0,T]}\times \Sigma . \end{aligned} \right. \end{aligned} \end{aligned}$$

In the coupled system (1)-(2), $c > 0$ is the wave speed and $\kappa > 0$ is the heat diffusion coefficient, the source terms are denoted by *f* and *g*, and $u_0$, $v_0$ and $\dot{v}_{0}$ are the initial conditions. On each external boundary $\Gamma _w$ and $\Gamma _h$, we have chosen a Robin condition with Robin parameters $\alpha _w$ and $\alpha _h$, so that by setting $\alpha _w = 0$, or $\alpha _w =+\infty$, we can also obtain Neumann and Dirichlet boundary conditions (and similarly for $\alpha _h$). The essential and natural transmission conditions on $\Sigma$ lead to a well-posed problem, with energy that remains bounded in time. They mimic the transmission of velocities and surface constraints (action-reaction principle) in the case of more realistic fluid-structure interaction problems, see [25, 45, 46].

## Heterogeneous OSWR

We now present and study a heterogeneous OSWR algorithm for the coupled heat-wave problem (1)-(2). The algorithm starts with an initial guess $u^0$ : ${[0,T]}\times \Omega _h\longrightarrow  \mathbb {R}$, which can be arbitrary, and then computes on both the heat and wave domain for iteration index $k=1,2,\ldots$

3 $$\begin{aligned} \begin{aligned}&\left\{ \begin{array}{rcll} \partial _t^2 v^k - c^2 \,\partial _x^2 v^k & =&  f, & \text {in}\ {[0,T]}\times \Omega _w, \\ v^k(0,\cdot ) & =&  v_0, & \text {in}\ \Omega _w, \\ \partial _tv^k(0,\cdot ) & =&  \dot{v}_0, & \text {in}\ \Omega _w, \\ -{\partial _{x}}v^k + \alpha _w \, v^k & =&  0, & \text {on}\ {[0,T]}\times \Gamma _w, \\ \left( S_1 \partial _t + c^2 \partial _x\right) v^k & =&  \left( S_1 + \kappa \partial _x\right) u^{k-1} &  \text {on}\ {[0,T]}\times \Sigma , \end{array} \right. \\&\left\{ \begin{array}{rcll} \partial _tu^k - \kappa \, \partial _x^2 u^k & =&  g, & \text {in}\ {[0,T]}\times \Omega _h, \\ u^k(0,\cdot ) & =&  u_0, & \text {in}\ \Omega _h, \\ {\partial _{x}}u^k + \alpha _h \, u^k & =&  0, & \text {on}\ {[0,T]}\times \Gamma _h, \\ \left( S_2 + \kappa \partial _x\right) u^k & =&  \left( S_2 \partial _t + c^2 \partial _x\right) v^{k}&  \text {on}\ {[0,T]} \times \Sigma , \end{array} \right. \end{aligned} \end{aligned}$$

where $S_1$ and $S_2$ are general operators to be chosen such that the convergence of the algorithm is fast. An alternating version can also be considered, by replacing $v^{k}$ in the last line on the right by $v^{k-1}$, the algorithm can then be executed in parallel. The convergence of both variants is very much related: in fact, the parallel version computes simultaneously two alternating iterations, starting once on the wave and once on the heat domain.

### Convergence analysis using laplace transforms

In order to study the heterogeneous OSWR algorithm (3) and optimize the transmission conditions, we consider now an unbounded time interval $T=\infty$ and use the Laplace transform in time with Laplace parameter $\tau \in \mathbb {C}$, $\mathcal{R}e(\tau )\ge 0$,

4 $$\begin{aligned} \begin{aligned}&\widetilde{ v}(x,\tau ) := \mathscr {L}_t( v)(x,\tau ) = \int _{\mathbb {R}^+} v(x,t) e^{-\tau t} \textrm{d}t, \\&\widetilde{u}(x,\tau ) := \mathscr {L}_t(u)(x,\tau ) = \int _{\mathbb {R}^+} u(x,t) e^{- \tau t} \textrm{d}t. \end{aligned} \end{aligned}$$

Since the problem is linear, we can directly study the error equations and set the source terms and initial conditions to zero, $f\equiv 0$, $g \equiv 0$, $v_0\equiv 0$, $\dot{v}_0\equiv 0$ and $u_0\equiv 0$. Applying the Laplace transform to the wave equation in (3) with $f\equiv 0$ gives

$$ \partial _x^2 \widetilde{ v}^k(x,\tau ) - \left( \frac{\tau }{c} \right) ^2 \widetilde{ v}^k(x,\tau ) = 0, \qquad x\in \Omega _w, $$

whose general solution is

5 $$\begin{aligned} \widetilde{ v}^k(x,\tau ) = A_w^k(\tau ) e^{\frac{\tau }{c}\,x} + B_w^k(\tau ) e^{-\frac{\tau }{c}\,x}, \qquad x\in \Omega _w. \end{aligned}$$

From the Robin boundary condition on the outer boundary $\Gamma _w$, which in Laplace space is

$$\begin{aligned} -{\partial _{x}}\widetilde{ v}^k(-l_w,\tau ) + \, \alpha _w \, \widetilde{ v}^k(-l_w,\tau ) = 0, \end{aligned}$$

we obtain $B_w^k(\tau ) = - A_w^k (\tau ) \frac{\alpha _w - \frac{\tau }{c}}{\alpha _w + \frac{\tau }{c}} e^{-2 \frac{\tau }{c}\,l_w}$, and thus the solution of the wave problem in Laplace space is of the form

$$ \widetilde{ v}^k(x,\tau ) = A_w^k(\tau ) \left( e^{\frac{\tau }{c}\,x} - \frac{\alpha _w - \frac{\tau }{c}}{\alpha _w + \frac{\tau }{c}} e^{\frac{\tau }{c}\, (-2 l_w - x)} \right) , \qquad x\in \Omega _w. $$

Its derivative in space can easily be computed to be

$$ \partial _x\widetilde{ v}^k(x,\tau ) = A_w^k(\tau ) \left( \frac{\tau }{c} e^{\frac{\tau }{c}\,x} + \frac{\tau }{c} \frac{\alpha _w - \frac{\tau }{c}}{\alpha _w + \frac{\tau }{c}} e^{\frac{\tau }{c}\, (-2 l_w - x)} \right) , \qquad x\in \Omega _w, $$

which can be rewritten in the form

6 $$\begin{aligned} \partial _x\widetilde{ v}^k(0,\tau ) = \phi _w(\tau ) \widetilde{ v}^k(0,\tau ) \text{ with } \phi _w(\tau ) := \frac{\tau }{c} \frac{1 + \frac{\alpha _w - \frac{\tau }{c}}{\alpha _w + \frac{\tau }{c}} e^{\frac{-2\tau l_w}{c} } }{1 - \frac{\alpha _w - \frac{\tau }{c}}{\alpha _w + \frac{\tau }{c}} e^{\frac{-2\tau l_w}{c}} }. \end{aligned}$$

We next consider the heat equation in (3), which with zero source term $g\equiv 0$ becomes in Laplace space

$$ \partial _x^2 \widetilde{u}^k(x,\tau ) - \, \frac{\tau }{\kappa } \, \widetilde{u}^k(x,\tau ) = 0, \qquad x\in \Omega _h. $$

The general solution is therefore of the form

7 $$\begin{aligned} \widetilde{u}^k(x,\tau ) = A_h^k(\tau ) e^{\sqrt{\frac{\tau }{\kappa }}\,x} + B_h^k(\tau ) e^{-\sqrt{\frac{\tau }{\kappa }}\,x}, \qquad x\in \Omega _h. \end{aligned}$$

The Robin boundary condition on the outer boundary $\Gamma _h$ becomes after the Laplace transform

8 $$\begin{aligned} {\partial _{x}}\widetilde{u}^k(l_h,\tau ) + \alpha _h \, \widetilde{u}^k(l_h,\tau ) = 0. \end{aligned}$$

Inserting (8) into equation (7), evaluated at $x=l_h$, yields $B_h^k(\tau ) \!=\! \frac{\sqrt{\frac{\tau }{\kappa }} + \alpha _h}{\sqrt{\frac{\tau }{\kappa }} - \alpha _h} A_h^k(\tau ) e^{\sqrt{\frac{\tau }{\kappa }}\, 2 l_h}$. As a result, we get

$$\begin{aligned} \widetilde{u}^k(x,\tau ) = A_h^k(\tau ) \left( e^{\sqrt{\frac{\tau }{\kappa }}\,x} + \frac{\sqrt{\frac{\tau }{\kappa }} + \alpha _h}{\sqrt{\frac{\tau }{\kappa }} - \alpha _h} e^{\sqrt{\frac{\tau }{\kappa }}\, (2 l_h-x)} \right) , \qquad x\in \Omega _h. \end{aligned}$$

The spatial derivative is readily computed to be

$$\begin{aligned} \partial _x\widetilde{u}^k(x,\tau ) = A_h^k(\tau ) \left( \sqrt{\frac{\tau }{\kappa }} e^{\sqrt{\frac{\tau }{\kappa }}\,x} - \sqrt{\frac{\tau }{\kappa }} \frac{\sqrt{\frac{\tau }{\kappa }} + \alpha _h}{\sqrt{\frac{\tau }{\kappa }} - \alpha _h} e^{\sqrt{\frac{\tau }{\kappa }}\, (2 l_h-x)} \right) , \qquad x\in \Omega _h, \end{aligned}$$

which can be written in the form

9 $$\begin{aligned} \partial _x\widetilde{u}^k(0,\tau ) = \phi _h(\tau ) \widetilde{u}^k(0,\tau ) \text{ with } \phi _h(\tau ) := \sqrt{\frac{\tau }{\kappa }} \, \frac{1 - \frac{\sqrt{\frac{\tau }{\kappa }} + \alpha _h}{\sqrt{\frac{\tau }{\kappa }} - \alpha _h} e^{ \sqrt{\frac{\tau }{\kappa }}\, (2 l_h)}}{1 + \frac{\sqrt{\frac{\tau }{\kappa }} + \alpha _h}{\sqrt{\frac{\tau }{\kappa }} - \alpha _h} e^{\sqrt{\frac{\tau }{\kappa }}\, (2 l_h)}}. \end{aligned}$$

From these computations, we can obtain a theoretically optimal choice of the operators $S_i$ in the transmission conditions for all time, $T=+\infty$:

#### Theorem 1

(Convergence Factor of heterogeneous OSWR) Let $s_i$, $i=1,2$, denote the Laplace symbols of $S_i$. The convergence factor of Algorithm (3) defined by

$\rho (\tau ;s_1,s_2):=\frac{\widetilde{ u}^k(0,\tau )}{\widetilde{ u}^{k-1}(0,\tau )}=\frac{\widetilde{ v}^k(0,\tau )}{\widetilde{ v}^{k-1}(0,\tau )}$ is given by

10 $$\begin{aligned} \rho ( \tau ; s_1 , s_2) := \rho _h ( \tau ; s_1, s_2)\, \rho _w ( \tau ; s_1, s_2), \end{aligned}$$

where the two factors are

11 $$\begin{aligned} \rho _h ( \tau ; s_1 , s_2 ) := \frac{s_1 + \kappa \phi _h (\tau )}{s_2 + \kappa \phi _h (\tau )} \end{aligned}$$

and

12 $$\begin{aligned} \rho _w ( \tau ; s_1 , s_2) := \frac{s_2 \tau + c^2 \phi _w (\tau )}{s_1 \tau + c^2 \phi _w(\tau )}. \end{aligned}$$

#### Proof

The transmission conditions in (3) in Laplace space are

$$ \begin{aligned}&( s_1 \tau + c^2 \partial _x) \widetilde{ v}^k(0,\tau ) = ( s_1 + \kappa \partial _x) \widetilde{u}^{k-1}(0,\tau ), \\&( s_2 + \kappa \partial _x) \widetilde{u}^k(0,\tau ) = ( s_2 \tau + c^2 \partial _x) \widetilde{ v}^{k}(0,\tau ). \end{aligned} $$

Using the explicit form of the wave and heat solution in (6) and (9) yields

$$ \begin{aligned}&( s_1\tau + c^2 \phi _w(\tau )) \widetilde{ v}^{k}(0,\tau )=(s_1+\kappa \phi _h(\tau ))\widetilde{u}^{k-1}(0,\tau ), \\&( s_2 + \kappa \phi _h(\tau ) ) \widetilde{u}^{k}(0,\tau )=(s_2\tau +c^2\phi _w(\tau ))\widetilde{ v}^{k}(0,\tau ). \end{aligned} $$

Combining the two equations then concludes the proof. $\square$

#### Remark 1

The alternating version of the heterogeneous OSWR achieves the convergence factor (10) in one alternating iteration, instead of two parallel ones.

#### Corollary 1

(Optimal choice of transmission operators) If $s_1=s_1^{\textrm{opt}}:=-\kappa \phi _h(\tau )$ and $s_2=s_2^{\textrm{opt}}:=-\frac{c^2}{\tau }\phi _w(\tau )$ then the algorithm converges in one iteration for all time, $t\in [0,T=+\infty )$. The corresponding transmission conditions are called the optimal transmission conditions.

#### Proof

The given $s_1^{\textrm{opt}}$ and $s_2^{\textrm{opt}}$ make the convergence factor vanish identically. $\square$

### Optimization of transmission conditions

The optimal operators corresponding to the Laplace symbols $s_1^{\textrm{opt}}$ and $s_2^{\textrm{opt}}$ are not differential operators, and would need convolution operations to be used, which is inconvenient and expensive in practice. We therefore follow the by now classical approach described in [2] to approximate the optimal choice and simply use constants $s_1$ and $s_2$, which are determined as solutions of a min–max problem obtained by setting $\tau :=i\omega$,

13 $$\begin{aligned} \inf _{(s_1,s_2)\in \mathbb {R}^2}\sup _{\omega \in [\omega _{\min },\omega _{\max }]} |\rho (i\omega ; s_1 , s_2)|, \end{aligned}$$

and the bounds on the frequency range can be estimated as $\omega _{\min }=\frac{\pi }{T}$, with *T* the length of the time interval used, and $\omega _{\max }=\frac{\pi }{\Delta t}$ with $\Delta t$ the time step, see e.g. [47]. Solving the min–max problem (13) is not straightforward for two parameters, and we therefore simplify now the problem further by reducing it to a one parameter min–max problem. A first idea is to choose

14 $$\begin{aligned} s_1 = -s_2 = s\in \mathbb {R}, \end{aligned}$$

and then to optimize using the one remaining parameter *s*. A second idea, inspired by [24], is to choose

15 $$\begin{aligned} s_2 = -c, \end{aligned}$$

and then to optimize using the remaining parameter $s_1$. In this approach, we impose the simple, transparent boundary condition for the wave equation on the unbounded domain, and use only the heat parameter $s_1$ to further optimize the convergence.

We analyze now the optimization for first choice (14), in which the convergence factor satisfies the following intriguing Lemma, which states that the wave domain does not contribute to the contraction of the algorithm.

#### Lemma 1

If $s_1 = -s_2 = s\in \mathbb {R}$, then the convergence factor of the Algorithm (3) satisfies

16 $$\begin{aligned} |\rho (i\omega ;s,-s)|=|\rho _h(i\omega ;s,-s)|. \end{aligned}$$

#### Proof

As we have shown in Theorem 1, the convergence factor is a product, $\rho =\rho _h\rho _w$, and we show now that if $s_1 = -s_2 = s\in \mathbb {R}$, then $|\rho _w|=1$. Using (12) we obtain

$$\begin{aligned} \rho _w ( i \omega ; s , -s) := \frac{-s i \omega + c^2 \phi _w (i\omega )}{s i\omega + c^2 \phi _w(i \omega )},\quad \text{ with }\quad \phi _w(i\omega ) = \frac{i\omega }{c} \frac{1 + \frac{\alpha _w - \frac{i\omega }{c}}{\alpha _w + \frac{i\omega }{c}} e^{\frac{i\omega }{c}\, (-2 l_w) } }{1 - \frac{\alpha _w - \frac{i\omega }{c}}{\alpha _w + \frac{i\omega }{c}} e^{\frac{i\omega }{c}\, (-2 l_w)} }. \end{aligned}$$

We next show that $\phi _w(i\omega )$ is a real number. To do so, we use that for any $a\in \mathbb {C}$ we have $\mathcal{R}e(\frac{1+a}{1-a})=\frac{1-|a|^2}{|1-a|^2}$ , which implies for our expression of $\phi _w(i\omega )$ with $\left| \frac{\alpha _w - \frac{i\omega }{c}}{\alpha _w + \frac{i\omega }{c}} e^{\frac{i\omega }{c}(-2 l_w)}\right| =1$ that the real part of the factor $\frac{1 + \frac{\alpha _w - \frac{i\omega }{c}}{\alpha _w + \frac{i\omega }{c}} e^{\frac{i\omega }{c}\, (-2 l_w) } }{1 - \frac{\alpha _w - \frac{i\omega }{c}}{\alpha _w + \frac{i\omega }{c}} e^{\frac{i\omega }{c}\, (-2 l_w)} }$ in $\phi _w(i\omega )$ must be 0, and hence $\phi _w(i\omega )$ is a real number.

This implies that the numerator and the denominator of $\rho _w ( i \omega ; s, -s)$ are conjugate, and hence $|\rho _w ( i \omega ; s, -s)|=1$ which concludes the proof. $\square$

With the choice (14), one can therefore only optimize the factor $|\rho _h|$ coming from the heat equation, and to simplify the resulting formulas, we assume that the heat domain is of infinite length here. This has very little influence on the resulting optimized parameter, the finite length of the wave domain is much more important, as we will see.

#### Theorem 2

(Optimized transmission parameter for choice 1) Let $l_h \rightarrow +\infty$. If $s_1=-s_2=s$, then the optimal parameter $s>0$ solving the min-max problem (13) is given by

17 $$\begin{aligned} s^\star =\sqrt{\kappa }(\omega _{\min }\omega _{\max })^{\frac{1}{4}}. \end{aligned}$$

Furthermore, with $\omega _{\max }=\pi /\Delta t$, the optimized parameter and corresponding convergence factor behave for $\Delta t$ small like

18 $$\begin{aligned} s^\star \sim \sqrt{\kappa }\frac{(\omega _{\min }\pi )^{\frac{1}{4}}}{\Delta t^{\frac{1}{4}}},\quad \inf _{s\in \mathbb {R}} \sup _{\omega \in [\omega _{\min },\omega _{\max }]}|\rho (i\omega ;s,-s)|^2\sim 1-2\sqrt{2}\left( \frac{\omega _{\min }}{\pi } \right) ^{\frac{1}{4}}\Delta t^{\frac{1}{4}}. \end{aligned}$$

#### Proof

With Lemma 1, it suffices to minimize $|\rho _h|$ defined in (11), and with the assumption $l_h \rightarrow +\infty$, the term $\phi _h(i\omega )$ becomes $\phi _h(i\omega ) = - \sqrt{\frac{i\omega }{\kappa }}$ and the convergence factor $\rho _h$ can be simplified to

$$\begin{aligned} \rho _h ( i \omega ; s , -s ) = \frac{s - \sqrt{ i w \kappa }}{-s - \sqrt{ i w \kappa }}\ \Longrightarrow \ | \rho _h( i \omega ; s , -s ) |^2 = \frac{s^2 - \sqrt{2 \omega \kappa }\, s + \omega \kappa }{s^2 + \sqrt{2 \omega \kappa }\, s + \omega \kappa }. \end{aligned}$$

To find the maximum of $|\rho _h|^2$ with respect to $\omega$ on an interval $[\omega _{\min },\omega _{\max }]$ we compute the derivative

$$ \frac{\partial }{\partial \omega } | \rho _h ( i \omega ; s , -s ) |^2 = \sqrt{\frac{2\kappa }{\omega }} \frac{ s ( \omega \kappa - s^2 )}{(s^2 + \sqrt{2 \omega \kappa }\, s + \omega \kappa )^2}. $$

**Fig. 2:**
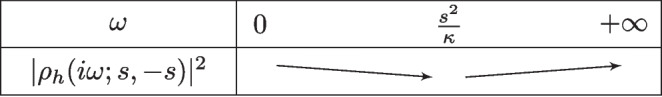
Variations of the function $\omega \rightarrow |\rho _h(i\omega ;s,-s)|^2$

This shows that the function $\omega \rightarrow |\rho _h(i \omega ; s , -s )|^2$ is first decreasing on $(0,\frac{s^2}{\kappa }]$ and then increasing on $[\frac{s^2}{\kappa },+\infty )$, see Fig. 2. We next consider the three possible positions of the interval $[\omega _{\min },\omega _{\max }]$ with respect to the inflection point $\frac{s^2}{\kappa }$, which shows that $\Phi (s) := \max _{\omega _{\min } \le \omega \le \omega _{\max }} |\rho _h (i\omega ; s , -s) |^2$ is given by

$$ \Phi (s) = \left\{ \begin{aligned}&|\rho _h (i\omega _{\max } ; s , -s) |^2  &   \text{ if } |s|\le \sqrt{\kappa \omega _{\min }}, \\&\max ( |\rho _h (i\omega _{\min } ; s , -s) |^2 , |\rho _h (i\omega _{\max } ; s , -s) |^2 )  &   \text{ if } \sqrt{\kappa \omega _{\min }}\le |s|\le \sqrt{\kappa \omega _{\max }}, \\&|\rho _h (i\omega _{\min } ; s , -s) |^2  &   \text{ if } |s|\ge \sqrt{\kappa \omega _{\max }}. \end{aligned} \right. $$

**Fig. 3:**
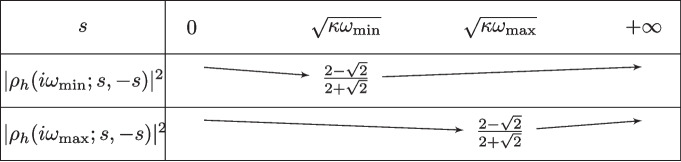
Variations of the functions $s\rightarrow |\rho _h(i\omega _{\min });s,-s)|^2$ and $s\rightarrow |\rho _h(i\omega _{\max };s,-s))|^2$

Now to find the minimum of $\Phi$ with respect to *s*, we compute the derivative

$$ \frac{\partial }{\partial s} | \rho _h(i \omega ; s , -s )|^2 = \frac{-2\sqrt{2\omega \kappa } ( \omega \kappa - s^2 )}{(s^2 + \sqrt{2 \omega \kappa }\, s + \omega \kappa )^2}, $$

which shows that the minimum of $s\rightarrow |\rho _h(i \omega ; s , -s )|^2$ is reached at $s=\sqrt{\kappa \omega }$. Hence for a given $\omega$, the function $s\rightarrow |\rho _h(i \omega ; s, -s )|^2$ is decreasing on $[0,\sqrt{\kappa \omega }]$ and increasing on $[\sqrt{\kappa \omega },+\infty )$. For any value of $\omega$ the minimum is $\frac{2-\sqrt{2}}{2+\sqrt{2}}$, see Fig. 3.

Thus we can compute the minimum of $\Phi$ on the three intervals:

- On $[\sqrt{\kappa \omega _{\min }},\sqrt{\kappa \omega _{\max }}]$ the minimum of $\Phi$ is reached when both functions $|\rho _h(i\omega _{\min };s,-s)|^2$ and $|\rho _h(i\omega _{\max };s,-s)|^2$ are equal, i.e. for $s^\star \in [\sqrt{\kappa \omega _{\min }},\sqrt{\kappa \omega _{\max }}]$ such that
  $$ |\rho _h (i\omega _{\min } ; s^* , -s^*) |^2 = |\rho _h (i\omega _{\max } ; s^* , -s^*) |^2. $$
  This leads after simplification to the equation
  $$ s(-2\sqrt{2}(\sqrt{\omega _{\min }\kappa }-\sqrt{\omega _{\max }\kappa })s^2-2\sqrt{2}\kappa (\omega _{\max }\sqrt{\kappa \omega _{\min }}-\omega _{\min }\sqrt{\kappa \omega _{\max }}))=0, $$
  whose unique positive root is (17), and then $\min _{\sqrt{\kappa \omega _{\min }}\le s\le \sqrt{\kappa \omega _{\max }}}\Phi (s)=|\rho _h (i\omega _{\max } ; s^\star , -s^\star ) |^2$.
- On $[0,\sqrt{\kappa \omega _{\min }}]$ we have $\min _{0\le s\le \sqrt{\kappa \omega _{\min }}}\Phi (s)\!=\!|\rho _h (i\omega _{\max };\! \sqrt{\kappa \omega _{\min }}, \!-\sqrt{\kappa \omega _{\min }}) |^2 \ge |\rho _h (i\omega _{\max } ; s^* , -s^*) |^2$.
- On $[\sqrt{\kappa \omega _{\max }},\!+\infty \!)$ we have $\min _{s\ge \sqrt{\kappa \omega _{\max }}}\Phi (s)\!=\!|\rho _h (i\omega _{\min };\! \sqrt{\kappa \omega _{\max }}, \!-\!\sqrt{\kappa \omega _{\max }}) |^2 \ge |\rho _h (i\omega _{\min } ; s^* , -s^*) |^2$.

This shows that the global minimum of $\Phi$ on $(0,+\infty )$ is reached at $s=s^\star$.

Now to find the behavior of the convergence factor when $\omega _{\max }=\frac{\pi }{\Delta t}$ and $\Delta t\rightarrow 0$, we expand for $\omega _{\max }$ tending to infinity to conclude the proof,

$$ |\rho _h(i\omega _{\min };s^\star ,-s^\star )|^2 =\frac{ \omega _{\min }^{1/2}\omega _{\max }^{1/2} - \sqrt{2} \omega _{\min }^{3/4}\omega _{\max }^{1/4} + \omega _{\min } }{ \omega _{\min }^{1/2}\omega _{\max }^{1/2} + \sqrt{2} \omega _{\min }^{3/4}\omega _{\max }^{1/4}+ \omega _{\min } }\simeq 1-2\sqrt{2}\omega _{\min }^{1/4}\omega _{\max }^{-1/4}. $$

$$\square $$

#### Remark 2

We see that with this first choice of the transmission parameters, the heterogeneous OSWR algorithm converges like OSWR applied to a decomposed heat equation problem [42], the wave equation does not contribute anything to the convergence of the method.

We next study the second choice, where we set $s_2:=-c$ and optimize the remaining parameter $s_1$ only. We first notice that the wave domain and the heat domain contribute very differently to the convergence of the iteration:

- the heat subdomain contributes for a good choice of the parameter $s_1$ to a uniform contraction over the entire time window [0, *T*].
- the wave subdomain contributes for a good choice of the parameter $s_2$ to convergence in a finite number of steps on a bounded time window [0, *T*].

In order to understand this convergence in a finite number of steps, suppose $s_2$ is chosen to obtain a transparent transmission conditions if the wave equation spatial domain was unbounded, i.e. $s_2:=-c$. Then, if the wave domain was really unbounded in space, one would achieve convergence in 3 parallel iterations, see [24]. In the case of a bounded wave domain in space and on a bounded time window [0, *T*], convergence is still in a finite number of iterations, as we show in the next theorem.

#### Theorem 3

(Finite Step Convergence of heterogeneous OWWR) If $s_2=-c$, and the time window length *T* satisfies $T\le kT_1$ with $T_1:=\frac{2|l_w|}{c}$, then convergence starting with the wave domain is achieved in at most *k* alternating iterations plus a final wave equation solve.

**Fig. 4:**
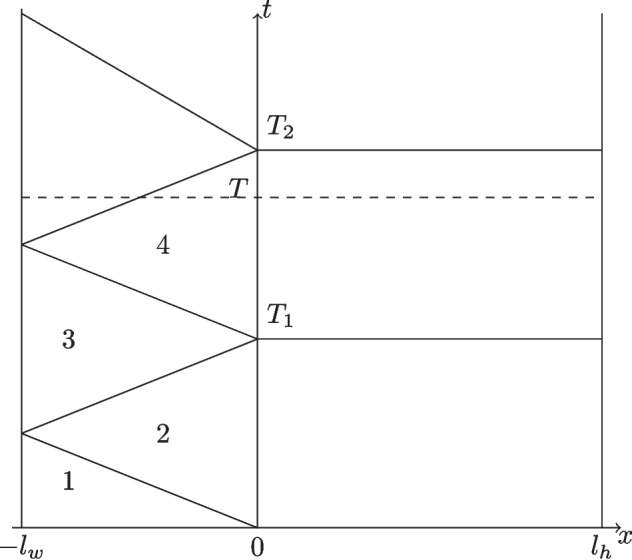
Convergence in a finite number of steps due to the wave domain when $s_2=-c$

#### Proof

Suppose we are interested in the solution up to time *T* indicated by the dashed line in Fig. 4, and we start by solving in the wave domain $\Omega _w$. Since the initial condition in $\Omega _w$ is known, and the outer boundary condition at $-l_w$ as well, the only error in this first solve on $\Omega _w$ is along the interface at $x=0$, over the entire time axis. Therefore the error is also zero in the lower left triangle marked with 1, below the first characteristic starting at (0, 0) with slope $-1/c$, since in this triangle the solution is entirely determined by the initial condition and the outer boundary condition, due to the finite speed of propagation *c* in the hyperbolic wave domain $\Omega _w$. In the triangle above this characteristic, marked with 2, the error in the solution of the wave equation is of the form $v^1(x,t)=g(x+ct)$, since only a left going wave can come from the error at the interface at $x=0$, and only above the second characteristic with slope 1/*c* there is also a right going component of the error, because of the reflection at the outer boundary at $x=-l_w$, provided a non-transparent boundary condition is imposed there. When we solve now on the heat domain $\Omega _h$, in the error equations the transmission condition

19 $$\begin{aligned} (s_2+\kappa \partial _x)u^1=(s_2\partial _t+c^2\partial _x) v^1 \end{aligned}$$

is imposed on $\Sigma$, with $u^1$ in the heat domain $\Omega _h$ and $v_1$ in the wave domain $\Omega _w$, and for $t\le T_1$ we obtain for the right hand side in (19)

$$ (s_2\partial _t+c^2\partial _x) v^1(x,t)=(s_2\partial _t+c^2\partial _x)g(x+ct)= s_2cg'(x+ct)+c^2g'(x+ct)=0, $$

since $s_2=-c$. Therefore, by the causality principle, the error in the heat solve on $\Omega _h$ is zero for $t\le T_1$, because on the initial line $t=0$, on the outer boundary at $x=l_h$ and also on the interface at $x=0$ the condition imposed on the error by (19) is zero (for $t\le T_1$). Solving again on the wave domain $\Omega _w$, we have now the correct data along the interface $x=0$ for $t\le T_1$, and thus the error is only non-zero for $t>T_1$, leading after the wave solve to the exact solution in the triangle 1 as before, but now also in triangles 2 and 3, and only a left going wave in triangle 4. Solving again on the heat domain $\Omega _h$, the solution is now correct for $t\le T_2$, and since $T\le T_2$, the algorithm has converged in $\Omega _h$. Solving again in the wave domain $\Omega _w$, we also have the exact solution there. The general result follows by induction in the same way. $\square$

The heterogeneous OSWR algorithm therefore converges with this second choice still in a finite number of steps, like for the unbounded wave domain, but if $T>T_1$, more iterations will be needed than for the unbounded wave domain case.

**Fig. 5:**
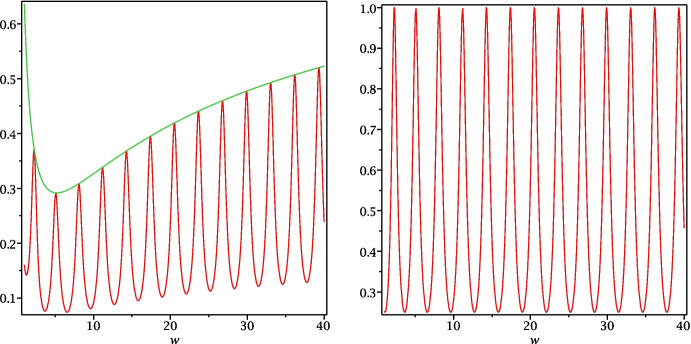
Case $s_2=-c$. Left: plot of $|\rho |^2$ and $|\rho _h|^2$ in the simplified situation where the heat domain is unbounded, as functions of $\tau =i\omega$. Right: corresponding plot for $|\rho _w|^2$

In this second choice, $s_2=-c$, we still have the parameter of the heat subdomain $s_1$ to optimize the performance of the heterogeneous OSWR algorithm, and as before, to simplify the formulas, we assume again that the heat domain is of infinite size, $l_h\rightarrow \infty$, since this has very little influence on the result, compared to the wave domain size, as we have now seen. We show in Fig. 5 on the left the convergence factor in modulus squared, $|\rho |^2$, and also $|\rho _h|^2$, for the parameter choice $\kappa =1$, $c=1$, $l_w=-1$, $a_w=2$, $s_1=2$ with $\tau =i\omega$. On the right, we show the corresponding result for $|\rho _w|^2$. We see that in the case^1^$s_1\ge c$, the oscillating convergence factor $|\rho |^2$ is bounded by the envelope function $|\rho _h|^2$, and hence we propose to study the approximate optimization problem

20 $$\begin{aligned} \min _{s_1\ge c}\max _{\omega \in [\omega _{\min },\omega _{\max }]}|\rho _h(i\omega ;s_1,-c)|^2. \end{aligned}$$

#### Theorem 4

Let $l_h\rightarrow +\infty$, $\alpha _h\rightarrow +\infty$ and $s_2=-c$, and assume that

21 $$\begin{aligned} \frac{\sqrt{\omega _{\min }}}{\sqrt{\omega _{\max }}}\le \frac{\sqrt{2\omega _{\min }\kappa }+c}{\sqrt{2\omega _{\min }\kappa }+2c}\quad \text{ and }\quad \kappa \sqrt{\omega _{\min }\omega _{\max }}-c^2>0. \end{aligned}$$

Then the solution of the min–max problem (20) is given by

22 $$\begin{aligned} s_1^\star =\frac{c(\sqrt{\omega _{\min }\kappa }+\sqrt{\omega _{\max }\kappa })+\sqrt{2}\kappa \sqrt{\omega _{\min }}\sqrt{\omega _{\max }}}{\sqrt{2}c+\sqrt{\omega _{\min }\kappa }+\sqrt{\omega _{\max }\kappa }}. \end{aligned}$$

Moreover if $\omega _{\max }:=\pi /\Delta t$, then the optimized parameter behaves for $\Delta t$ small like

23 $$\begin{aligned} s_1^*\sim c+\sqrt{2\kappa \omega _{\min }} -\sqrt{2}\frac{c\sqrt{2\kappa \omega _{\min }}+c^2+\kappa \omega _{\min }}{\sqrt{\kappa \pi }}\sqrt{\Delta t} \end{aligned}$$

and the associated asymptotic convergence factor satisfies

24 $$\begin{aligned} \min _{s_1\ge 0}\max _{\omega \in [\omega _{\min },\omega _{\max }]}|\rho _h|^2(i\omega ;s_1,-c)\sim 1-\frac{\sqrt{2}(2c+\sqrt{2\kappa \omega _{\min }})}{\sqrt{\pi \kappa }}\sqrt{\Delta t}. \end{aligned}$$

#### Proof

When $l_h\rightarrow +\infty$ and $\alpha _h\rightarrow +\infty$, using the definitions (9) and (11), we get $\rho _h(\tau ;s_1,-c)=\frac{\sqrt{\kappa \tau }-s_1}{\sqrt{\kappa \tau }+c}$, and we have for $\tau =i\omega$

$$ |\rho _h|^2(i\omega ;s_1,-c)= \frac{s_1^2-\sqrt{2\omega \kappa }s_1+\omega \kappa }{c^2 +\sqrt{2\omega \kappa }c+\omega \kappa }. $$

To solve (20), we first study the behavior of the function $\omega \rightarrow |\rho _h|^2(i\omega ;s_1,-c)$ for a fixed $s_1$. Computing the derivative gives

$$ \frac{\partial |\rho _h|^2}{\partial \omega }(i\omega ;s_1,-c) =\frac{\kappa (c+s_1)}{2\sqrt{\omega \kappa }}\frac{2(c-s_1)\sqrt{\omega \kappa }+\sqrt{2}\omega \kappa -\sqrt{2}s_1c}{(c^2 +\sqrt{2\omega \kappa }c+\omega \kappa )^2}, $$

which shows that $\omega \rightarrow |\rho _h({i\omega ;s_1,-c})|^2$ is decreasing on $(0,\omega _0(s_1))$ and increasing on $(\omega _0(s_1),+\infty )$ where $\omega _0(s_1)=(\sqrt{c^2+s_1^2}-(c-s_1))^2/2\kappa$. Therefore, $\Phi (s_1):=\max _{\omega \in [\omega _{\min },\omega _{\max }]} |\rho _h(i\omega ;s_1,-c)|^2$ is given by

$$ \Phi (s_1)= \left\{ \begin{aligned}&|\rho _h(i\omega _{\min };s_1,-c)|^2  &   \text{ if } \omega _{\max }\le \omega _0(s_1), \\&{\max (|\rho _h(i\omega _{\min };s_1,-c)|^2,|\rho _h(i\omega _{\max };s_1,-c)|^2)}  &   \text{ if } \omega _{\min }\le \omega _0(s_1) \le \omega _{\max }, \\&|\rho _h(i\omega _{\max };s_1,-c)|^2  &   \text{ if } \omega _0(s_1)\le \omega _{\min }, \end{aligned} \right. $$

or equivalently, since the function $s_1\rightarrow \omega _0(s_1)$ is increasing,

$$ \Phi (s_1)= \left\{ \begin{aligned}&|\rho _h(i\omega _{\max };s_1,-c)|^2  &   \text{ if } s_1\le s_1^{\min }, \\&{\max (|\rho _h(i\omega _{\min };s_1,-c)|^2,|\rho _h(i\omega _{\max };s_1,-c)|^2)}  &   \text{ if } s_1^{\min }\le s_1 \le s_1^{\max }, \\&|\rho _h(i\omega _{\min };s_1,-c)|^2  &   \text{ if } s_1\ge s_1^{\max }, \end{aligned} \right. $$

where we have introduced $s_1^{\min }$ and $s_1^{\max }$, the solutions of $\omega _0(s_1^{\min })=\omega _{\min }$ and $\omega _0(s_1^{\max })=\omega _{\max }$, which are

$$ \begin{aligned}&s_1^{\min }:=\frac{\omega _{\min } \kappa +c\sqrt{2\omega _{\min } \kappa }}{\sqrt{2\omega _{\min } \kappa }+c}=\frac{\sqrt{\omega _{\min }\kappa }}{\sqrt{2}}\frac{\sqrt{2\omega _{\min }\kappa }+2c}{\sqrt{2\omega _{\min }\kappa }+c}, \\&s_1^{\max }:=\frac{\sqrt{\omega _{\max }\kappa }}{\sqrt{2}}\frac{\sqrt{2\omega _{\max }\kappa }+2c}{\sqrt{2\omega _{\max }\kappa }+c}. \end{aligned} $$

With the first assumption in (21), we have the inequalities

$$ \sqrt{\frac{\omega _{\min } \kappa }{2}}\le s_1^{\min }\le \sqrt{\frac{\omega _{\max } \kappa }{2}} \le s_1^{\max }. $$

**Fig. 6:**
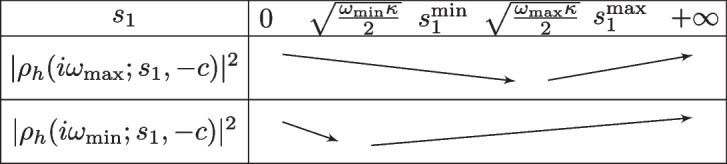
Variations of $s_1\rightarrow |\rho _h(i\omega _{\min };s_1,-c)|^2$ and $s_1\rightarrow |\rho _h(i\omega _{\max };s_1,-c)|^2$

Moreover, for a fixed $\omega$, the function $s_1\rightarrow |\rho _h|^2({i\omega ;s_1,-c})$ is a second degree polynomial, and it is decreasing on $[0,\sqrt{\frac{\omega \kappa }{2}})$ and increasing on $(\sqrt{\frac{\omega \kappa }{2}},+\infty )$, see Fig. 6, which implies

**Fig. 7:**
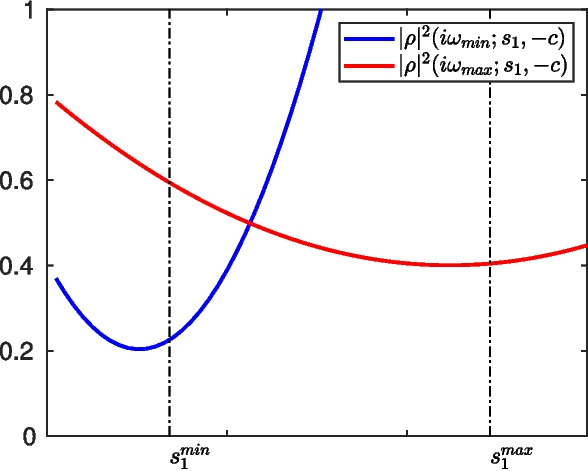
Example of $|\rho _h(i\omega _{\min };s_1,-c)|^2$ and $|\rho _h(i\omega _{\max };s_1,-c)|^2$ as functions of $s_1$

- on $[0,s_1^{\min }]$, since $s_1^{\min }\le \sqrt{\frac{\omega _{\max } \kappa }{2}}$ that
  $$ \min _{s\le s_1^{\min }}\Phi (s_1)=\min _{s\le s_1^{\min }}|\rho _h(i\omega _{\max };s_1,-c)|^2 =|\rho _h(i\omega _{\max };s_1^{\min },-c)|^2; $$
- on $[s_1^{\max },+\infty )$, since $\sqrt{\frac{\omega _{\min } \kappa }{2}}\le s_1^{\max }$ that
  $$ \min _{s\ge s_1^{\max }}\Phi (s_1)=\min _{s\ge s_1^{\max }}|\rho _h(i\omega _{\min };s_1,-c)|^2=|\rho _h(i\omega _{\min };s_1^{\max },-c)|^2; $$
- and on $[s_1^{\min },s_1^{\max }]$, since the function $\omega \rightarrow |\rho _h(i\omega ;s_1^{\min },-c)|^2$ is increasing on $[\omega _{\min },\omega _{\max }]$ that $|\rho _h(i\omega _{\min };s_1^{\min },-c)|^2\le |\rho _h(i\omega _{\max };s_1^{\min },-c)|^2$. In the same way, we have if $|\rho _h(i\omega _{\min };s_1^{\max },-c)|^2\ge |\rho _h(i\omega _{\max };s_1^{\max },-c)|^2$ that the minimum of $\Phi$ is reached at $s_1=s_1^\star$ with $|\rho _h|^2(s_1^\star ;\omega _{\min },-c)=|\rho _h|^2(s_1^\star ;\omega _{\max },-c)$, see Fig. 7. Simplifying this equation, we find that $s_1^{\star }$ is solution of
  $$\begin{aligned}&s^2(\sqrt{2}c+\sqrt{\omega _{\min }\kappa }+\sqrt{\omega _{\max }\kappa }) +s\sqrt{2}(c^2-\kappa \sqrt{\omega _{\min }}\sqrt{\omega _{\max }})\\&\quad -c(c(\sqrt{\omega _{\min }\kappa }+\sqrt{\omega _{\max }\kappa })+\sqrt{2}\kappa \sqrt{\omega _{\min }}\sqrt{\omega _{\max }}))=0. \end{aligned}$$
  This equation has two roots, one equals $-c<0$, and the other one is shown in (22).

Hence $s^\star$ in (22) is the solution, provided it is larger than *c*. A direct verification shows that this is the case if the second inequality in (21) holds. This is true when $\omega _{\max }$ is large, i.e. $\Delta t$ small, for which we obtain (23) and (24), which concludes the proof. $\square$

#### Remark 3

The optimized parameter $s_1^*$ from Theorem 4 is very different from the optimized parameter one obtains for example when coupling two heat equations, since it tends to a constant, while for two heat equations, it grows like $\Delta t^{-1/4}$, see [42]. This is because in the denominator of $\rho _h$ the parameter is fixed to *c*, while in the heat equation case, it would also equal $s_1$. Also, the asymptotic contraction factor in our heterogeneous case behaves like $1-O(\sqrt{\Delta t})$, and when coupling two heat equations it is $1-O(\Delta t^{1/4})$, see [42].

Note that we can obtain a slightly better estimate, if we do not equioscillate between $\omega _{\min }$ and $\omega _{\max }$, but really between the first and last point where the maximum is actually attained by $|\rho |$, as seen in Fig. 5 on the left. The location of these maxima can be estimated by a direct calculation, as one can suspect from the regularity of the oscillating function in Fig. 5 on the right. Computing the derivative of $|\rho _w|^2$ with respect to $\omega$, we find that the locations of the extrema are given by

$$ \bar{\omega }=\frac{cz}{2l_w}, $$

where *z* is solution of the transcendental equation

$$ f(z):=\tan (z)(z^2-4\alpha _w^2l_w^2)+4zl_w\alpha _w=0. $$

Choosing instead of $\omega _{\min }$ in the min-max problem (20) the corresponding first maximum point $\bar{\omega }$ (which is larger than $\omega _{\min }$), and instead of $\omega _{\max }$ in the min-max problem (20) the last maximum point $\bar{\omega }$ (which is smaller than $\omega _{\max }$), equioscillation would give then the truly best possible parameter $s_1^*$ one could use. Note that this parameter would behave asymptotically as predicted by Theorem 4. Moreover, one could use the formula (22) in Theorem 4 replacing $\omega _{\min }$ by the corresponding first maximum point $\bar{\omega }$ (larger than $\omega _{\min }$) to obtain an asymptotic formula for the truly best possible parameter $s_1^*$.

## Numerical experiments

We present now numerical experiments, both for a monolithic scheme, as in [24], and then also for OSWR, with the two choices of optimization from Subsection 3.2.

**Fig. 8:**
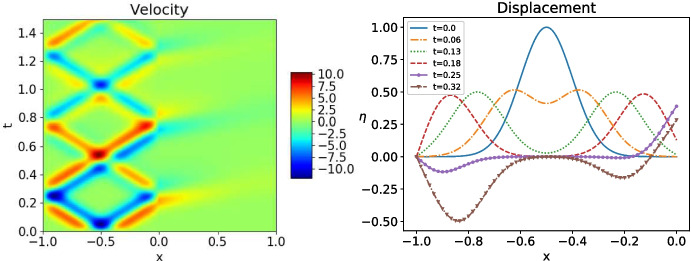
Initial data supported in the wave domain. Left: monolithic numerical approximation for the velocity $\partial _t v$ in $\Omega _w$ and *u* in $\Omega _h$. Right: displacement *v* in $\Omega _w$ at several points in time

### The monolithic solution

We use a Crank-Nicolson scheme to numerically solve the heat-wave coupled problem (1). We discretize $$[-l_w,l_h]$$ for the case ł$$_w=l_h$$ using the uniform mesh $$(x_i)_{-N\le i\le N}$$ with mesh size $$\Delta x$$, and $$\Delta t$$ is the time step. The scheme for the wave equation for $$-N+1\le j\le -1$$ with $$\xi _j^{n+1/2}:=( v_{j}^{n+1}- v_j^n)/\Delta t$$ and $$ v^{n+1/2}_j:=( v_j^{n+1}+ v_j^n)/2$$ is

$$ \frac{\xi _{j}^{n+1}-\xi _{j}^n}{\Delta t}-\frac{1}{\Delta x}\left( c^2\frac{ v_{j+1}^{n+1/2}- v_j^{n+1/2}}{\Delta x}-c^2\frac{ v_{j}^{n+1/2}- v_{j-1}^{n+1/2}}{\Delta x}\right) =f(x_j,t^{n+1/2}). $$

The heat equation scheme for $1\le j\le N-1$ is

$$ \frac{u_j^{n+1}-u_j^n}{\Delta t}-\frac{1}{\Delta x}\left( \kappa \frac{u_{j+1}^{n+1/2}-u_j^{n+1/2}}{\Delta x}-\kappa \frac{u_{j}^{n+1/2}-u_{j-1}^{n+1/2}}{\Delta x}\right) =g(x_j,t^{n+1/2}). $$

At the interface, using the coupling conditions (2), we impose

$$ \begin{aligned}&\frac{\xi _{0}^{n+1}-\xi _{0}^n}{\Delta t}-\frac{2}{\Delta x}\left( -c^2\frac{ v_{0}^{n+1/2}- v_{-1}^{n+1/2}}{\Delta x}\right) +\frac{u_0^{n+1}-u_0^n}{\Delta t}-\frac{2}{\Delta x}\left( \kappa \frac{u_{1}^{n+1/2}-u_0^{n+1/2}}{\Delta x}\right) \\&\qquad =f(x_0,t^{n+1/2})+g(x_0,t^{n+1/2}),\quad \text{ with }\quad \xi _0^{n+1/2}=\frac{ v_{0}^{n+1}- v_0^n}{\Delta t}=u_0^{n+1/2}. \end{aligned} $$

We choose for the physical parameters $c=2$, $\kappa =1$, for the boundary conditions $\alpha _h=0$, $\alpha _w=+\infty$, for the domain sizes $l_w=l_h=1$ and $T=1.5$, and for the mesh sizes $\Delta x=\Delta t=\frac{2}{600}$. We show in Fig. 8 a typical test for $f=0$, $g=0$, $\dot{ v}_{0}=0$, and $( v_0,u_0)(x)=e^{-50(x+0.5)^2}$, i.e. the initial data is mostly supported in the wave domain. We clearly see the waves in $\Omega _w$ propagating at the speed $\frac{1}{c}$, and how a part of the information is transmitted to the heat domain and diffuses into it.

**Fig. 9:**
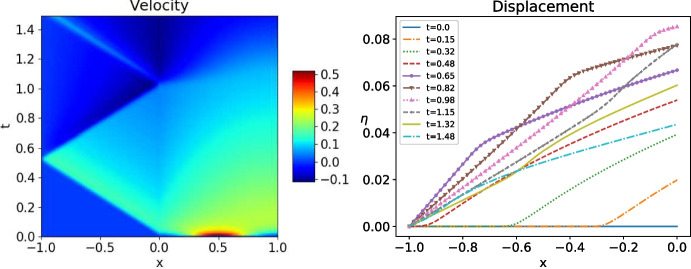
As in Fig. 8, but now with initial data supported in the heat domain

**Fig. 10:**
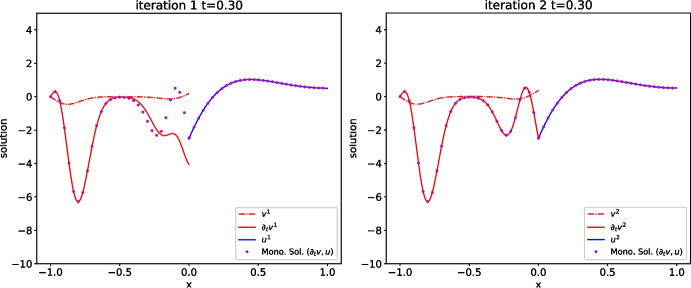
Discretized OSWR algorithm (3) with data in the wave domain: solution at $t=0.3$ after one iteration (left) and two iterations (right). Here $s_2=-c$ and $s_1=4$

In Fig. 9, we show the case $( v_0,u_0)(x)=e^{-50(x-0.5)^2}$, i.e. when the initial data is supported in the heat domain. The Gaussian is diffused and when it reaches the wave domain it is transported at the speed $\frac{1}{c}$, and reflections appear at $x=-1$, c.f. Theorem 3.

**Fig. 11:**
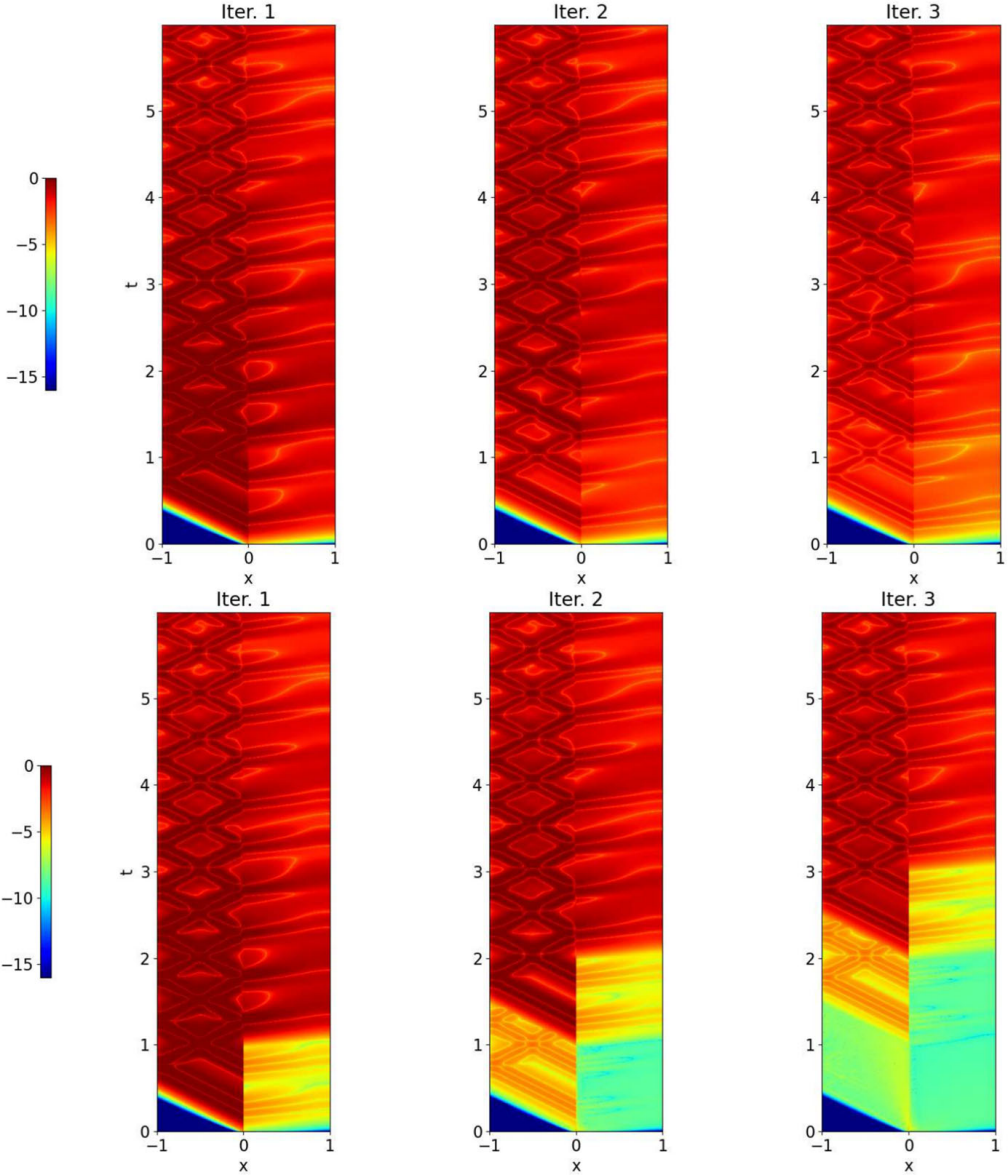
Discretized OSWR algorithm (3) with initial data in the wave domain: error after one, two and three iterations. Top: $s_1=-s_2=3.5$, bottom: $s_1=3,s_2=-c=-2$

**Fig. 12:**
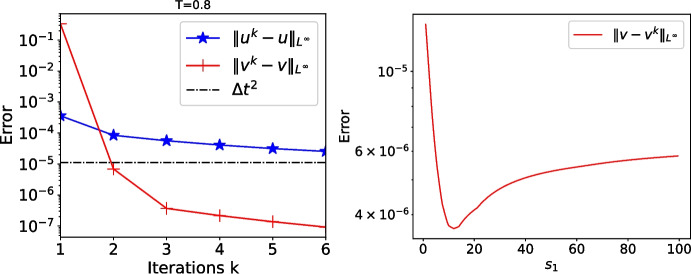
Errors when $s_2=-c$ for short time $T=0.8$ (in black truncation error levels of the scheme). Left: convergence with $s_1=4$. Right: error after 2 iterations as function of $s_1$

**Fig. 13:**
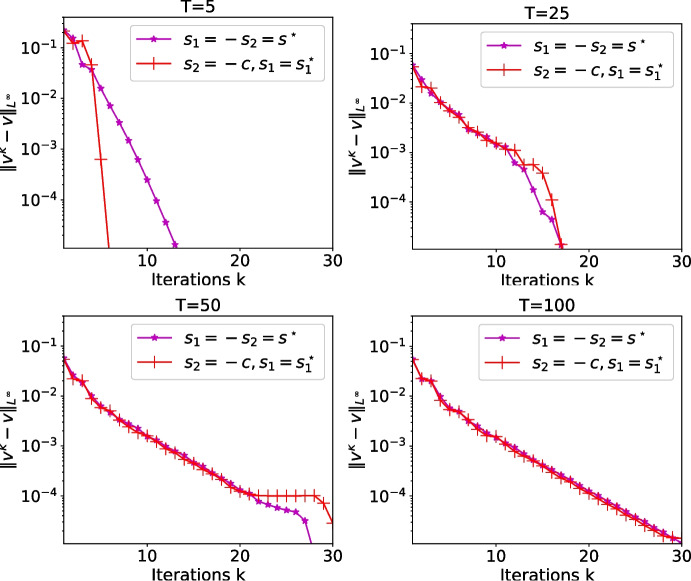
Errors for longer time $T=5,\,25,\,50$ and 100 with $s_1=-s_2=s^\star$ from the first optimization choice and $s_2=-c$ and $s_1=s_1^\star$ from the second optimization choice

### Illustration of heterogeneous OSWR

We first use the discretized heterogeneous OSWR algorithm (3) when $( v_0,u_0)(x)=e^{-50(x+0.5)^2}$, i.e. the initial data is in the wave domain, and $s_1=4$, $s_2=-c=-2$. In Fig. 10 we show the solution at $t=0.3$ for iterations 1 and 2. While at the first iteration the velocity is clearly not continuous at the interface, after only one more iteration the continuity is greatly improved. In Fig. 11 we show the error between the OSWR approximations at iterations 1, 2 and 3 and the monolithic solution on the time interval [0, 6]; at the top the case $s_1=-s_2=3.5$, at the bottom the case $s_2=-c=-2$, $s_1=3$. The case at the bottom illustrates well the convergence mechanisms analyzed in Theorem 3: we see in the first iteration in the wave domain on the left the typical triangle where there is no error due to the finite speed of propagation, and then in the second iteration a great error reduction in the rhomboid above due to the small error in the heat domain, and the reduction continues like this in the third iteration.

**Fig. 14:**
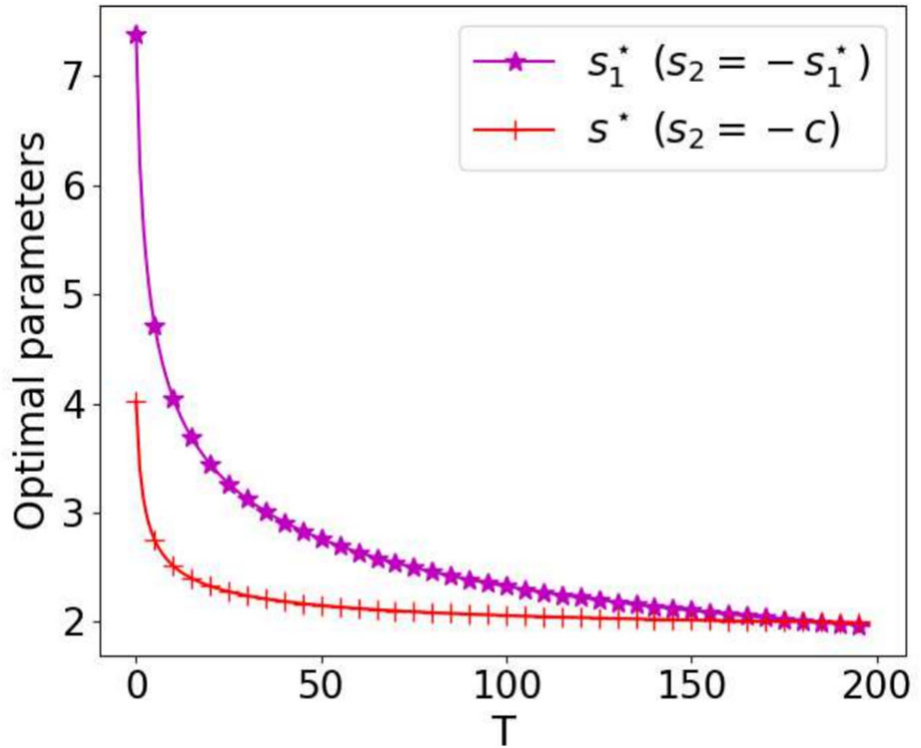
Optimal parameters from Theorems 2 and 4 as functions of *T*

**Fig. 15:**
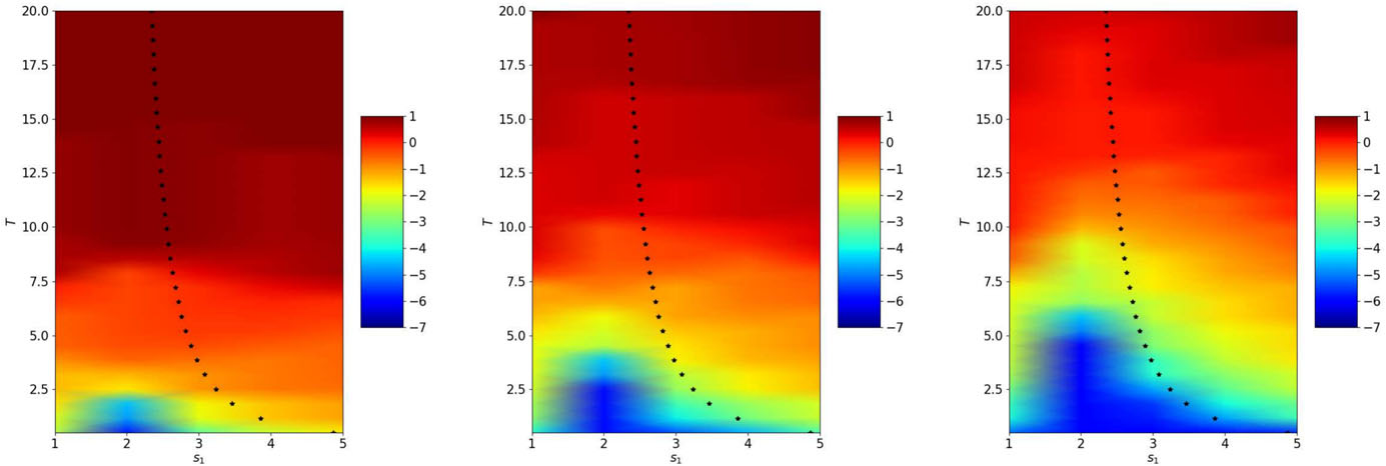
From left to right: logarithm of the error in the wave domain after 2, 4 and 6 iterations depending on $s_1=-s_2$ and *T*. The crosses represent the theoretical values $s^\star$ from Theorem 2

### Thorough numerical investigation of heterogeneous OSWR

We now study more precisely the numerical convergence of the algorithm. The physical data *f*, *g*, $v_0$, $\dot{v}_0$ and $u_0$ are now 0, the only non zero data is the initial guess used to start the iteration,

$$ (s_1+\kappa \partial _x)u^0=(s_1+\kappa )\frac{\sum _{j=1}^{100} t\sin (jt)}{\max _{t\in [0,T]}|\sum _{j=1}^{100} t\sin (jt)|}, $$

so that the want to compute the zero solution, and computing the norm of the solution is equivalent to compute the error in the algorithm. We first consider a short time interval $T=0.8<T_1=\frac{2|l_w|}{c}=1$ when $s_2=-c$. We show in Fig. 12 on the left the convergence history given by the errors $\Vert v-v^k\Vert _{L^\infty ({[0,T]}\times \Omega _w)}$ and $\Vert u-u^k\Vert _{L^\infty ({[0,T]}\times \Omega _h)}$ as functions of the iterations *k* (*u* and *v* are the monolithic solutions) when $s_1=4$. As predicted by Theorem 3, we obtain the solution (up to the truncation error of the scheme) after the second iteration, and other values of $s_1$ give similar results, as shown in Fig. 12 on the right.

**Fig. 16:**
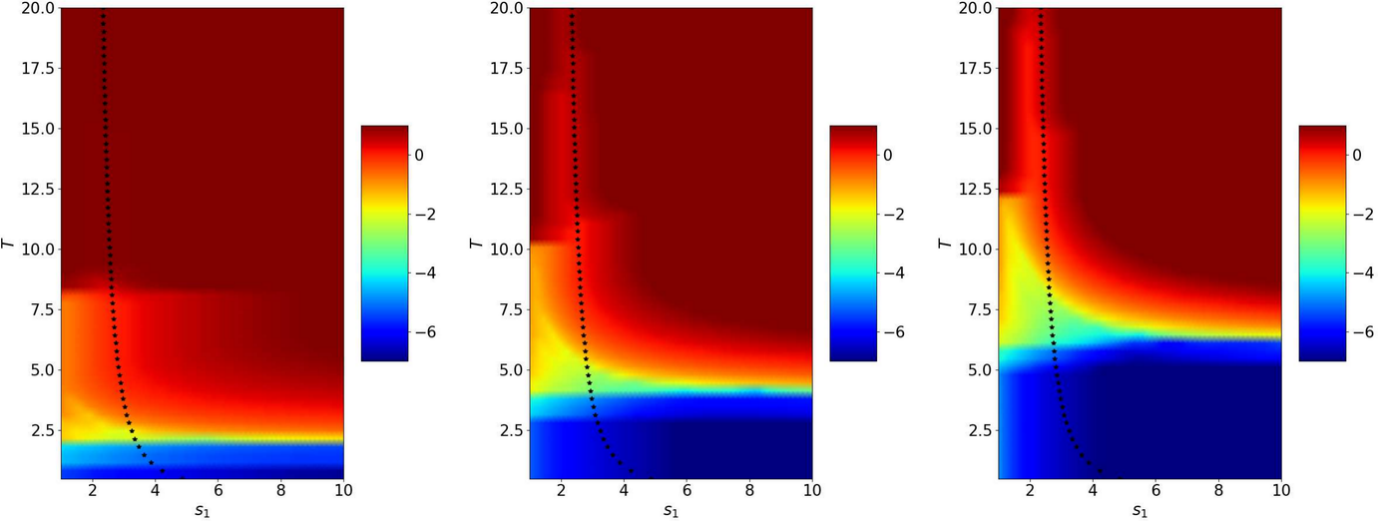
From left to right: logarithm of the error in the wave domain after 2, 4 and 6 iterations depending on $s_1$ and *T* when $s_2=-c$. The crosses represent the theoretical values $s_1^\star$ from Theorem 4

**Fig. 17:**
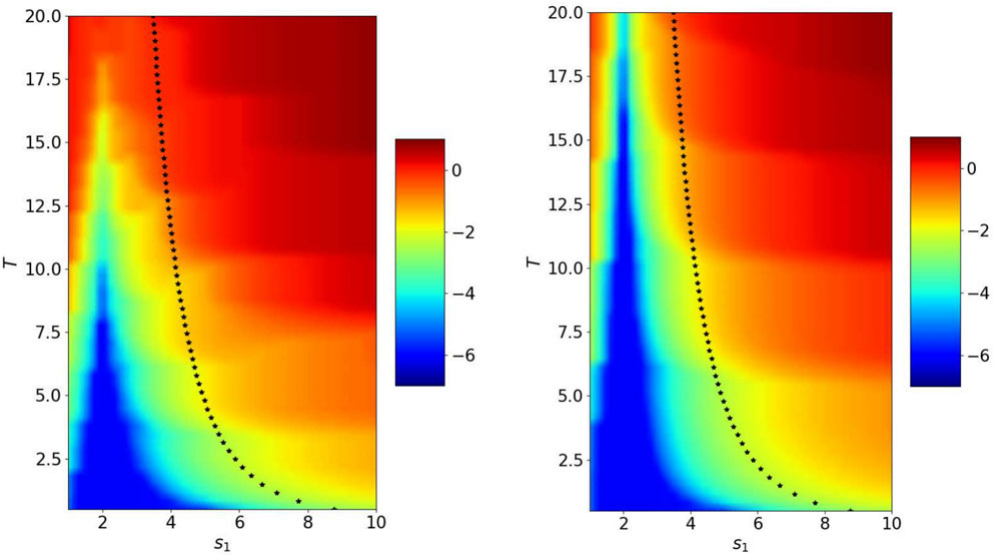
Logarithm of the error in the wave domain after 6 iterations depending on $s_1$ and *T* when $s_2=-s_1$ and $|l_w|=|l_h|=2$ and 4 (from left to right). The crosses represent the theoretical values $s^\star$ from Theorem 2

For larger values $T>T_1$, we show in Fig. 13 the convergence history of OSWR, when $s_1=-s_2=s^\star$ from the first optimization choice (Theorem 2) and with $s_2=-c$ and $s_1=s_1^\star$ from the second optimization choice (Theorem 4). We see that for the second choice and for longer time windows, convergence is not reached anymore after 2 iterations, as expected from our analysis, and now the value of the parameters has an important influence on the convergence speed.

To go further, we show in Fig. 14 the optimal parameters from Theorems 2 and 4 as functions of *T*. We observe that for the first choice, inspite of the fact that the limit for $T\rightarrow +\infty$ of $s_1^\star$ is 0, the convergence in $T^{-1/4}$ is slow and for $T=200$ this parameter is still close to $s^\star$ from the second choice. However in numerical simulations one typically considers small time intervals (if not, one should decompose the time interval into several time windows), and then we recommend to choose $s_2=-c$ to achieve the best convergence speed.

Figure 15 shows the error reached after 2, 4 and 6 iterations for $s_2=-s_1$ depending on $s_1$ and *T*. We see that when the number of iterations becomes larger, the optimized parameter from Theorem 2 behaves like the best performing numerical one. In Fig. 16 we show the error after 2, 4 and 6 iterations for $s_2=-c$. In the bottom part, we clearly see the behavior of the error described in Theorem 3 that convergence is reached in a finite number of iterations for a given *T*, but we also see above that the optimized parameter computed in Theorem 4 is close to the numerically best performing one.

**Fig. 18:**
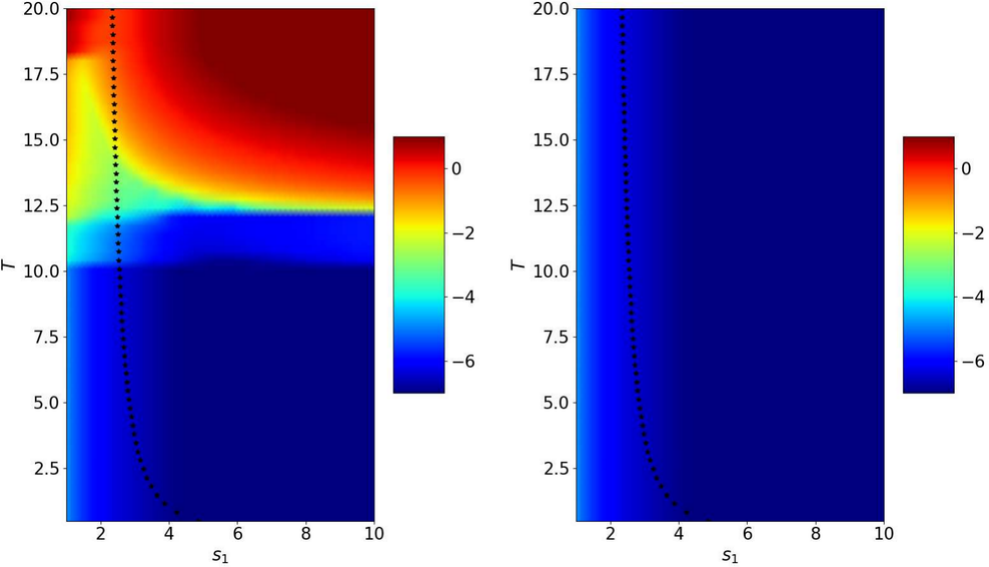
Logarithm of the error in the wave domain after 6 iterations depending on $s_1$ and *T* when $s_2=-c$ and $|l_w|=|l_h|=2$ and 4 (from left to right). The crosses represent the theoretical values $s_1^\star$ from Theorem 4

We now study the influence of $|l_w|=|l_h|$ in Figs. 17 and 18. As expected, the larger the domain length $|l_w|=|l_h|$, the better the computed parameter $s^\star$ in the case $s_2=-s_1$. We also see that the algorithm converges very quickly when $s_2=-c$.

## Conclusion

We made an important step forward in the design and analysis of time dependent fluid-structure interaction problems by studying as a first example of relevance a heterogeneous optimized Schwarz waveform relaxation algorithm applied to a heat-wave coupled problem in space time on bounded domains. We optimized two choices of transmission conditions, and showed that the boundedness of the wave domain has an important influence on the convergence mechanisms and the optimized choice of parameters. We illustrated our analysis with numerical experiments, which indicate that the second choice $s_2=-c$ is preferable, since it gives a very accurate solution for small time intervals (for larger time intervals one can use time windows). There are many further directions that need to be explored: higher spatial dimensions, more than two subdomains for layered material situations, the real fluid-structure interaction problem, and also more sophisticated analysis techniques to replace the Laplace transform well suited for long time intervals.

## Data Availability

No datasets were generated or analysed during the current study.

## Footnotes

1 If $s_1<c$, the oscillations are above the envelope and the algorithm can even diverge, because the convergence factor $\rho$ can exeed one.
